# Environmental Adaptation: Genomic Analysis of the Piezotolerant and Psychrotolerant Deep-Sea Iron Reducing Bacterium *Shewanella piezotolerans* WP3

**DOI:** 10.1371/journal.pone.0001937

**Published:** 2008-04-09

**Authors:** Fengping Wang, Jianbin Wang, Huahua Jian, Bing Zhang, Shengkang Li, Feng Wang, Xiaowei Zeng, Lei Gao, Douglas Hoyt Bartlett, Jun Yu, Songnian Hu, Xiang Xiao

**Affiliations:** 1 Key Laboratory of Marine Biogenetic Resources, State Oceanic Administration, Xiamen, People's Republic of China; 2 Third Institute of Oceanography, State Oceanic Administration, Xiamen, People's Republic of China; 3 Beijing Institute of Genomics, Chinese Academy of Sciences, Beijing, People's Republic of China; 4 James D. Watson Institute of Genome Sciences, Zhejiang University, Hangzhou, People's Republic of China; 5 The T-Life Research Center, Fudan University, Shanghai, People's Republic of China; 6 Center for Marine Biotechnology and Biomedicine, Marine Biology Research Division, Scripps Institution of Oceanography, University of California, San Diego, La Jolla, California, United States of America; Baylor College of Medicine, United States of America

## Abstract

*Shewanella* species are widespread in various environments. Here, the genome sequence of *Shewanella piezotolerans* WP3, a piezotolerant and psychrotolerant iron reducing bacterium from deep-sea sediment was determined with related functional analysis to study its environmental adaptation mechanisms. The genome of WP3 consists of 5,396,476 base pairs (bp) with 4,944 open reading frames (ORFs). It possesses numerous genes or gene clusters which help it to cope with extreme living conditions such as genes for two sets of flagellum systems, structural RNA modification, eicosapentaenoic acid (EPA) biosynthesis and osmolyte transport and synthesis. And WP3 contains 55 open reading frames encoding putative *c*-type cytochromes which are substantial to its wide environmental adaptation ability. The *mtr-omc* gene cluster involved in the insoluble metal reduction in the *Shewanella* genus was identified and compared. The two sets of flagellum systems were found to be differentially regulated under low temperature and high pressure; the lateral flagellum system was found essential for its motility and living at low temperature.

## Introduction

The deep sea, which is characterized by extremely low temperatures (<5°C) and high pressures (up to 110 MPa), comprises the bulk of the world's oceans. Microorganisms are known to thrive in this extreme environment, however, knowledge of the adaptations of deep-sea microorganisms to the psychrosphere/piezosphere remains fragmentary [1], [2], [3]. Recently genome sequence information of bacteria from high pressure and/or cold environments (including *Photobacterium profundum* [4], *Colwellia psychrerythraea* [5], *Idiomarina loihiensis* [6] and *Pseudoalteromonas haloplanktis* [7]) has indicated some of the relevant adaptation strategies. However, much more information is needed.

Members of the genus *Shewanella* inhabit various environments, and are well known for their versatile respiratory capabilities, coupling the oxidation of diverse substrates to the respiration of many different electron acceptors [8]. As a result of these properties, *Shewanella* strains have been proposed as candidates for bioremediation of metal and organic contaminants. Since potential clean up sites often include cold subsurface settings, it is essential to understand how these conditions influence *Shewanella* evolution, adaptation and ecology. One *Shewanella* species, *Shewanella oneidensis* MR-1, isolated from Oneida Lake sediments (NY, USA) [9] has been the subject of detailed genomic and proteomic investigations. The genome sequence of MR-1 has provided invaluable information about its respiratory capabilities [10], [11]. However, still little is known about environmental acclimation or evolution within this genus [12], [13]. At present, there are more than 18 *Shewanella* strains from various environments that have been sequenced by the Joint Genome Institute and other organizations [10], [14]. These sequences provide a powerful frame of reference for additional genomic analyses of new *Shewanella* isolates possessing distinctive phenotypes.

*Shewanella* strains are frequently the most abundant *Proteobacteria* in the benthos [15], [16], and in some deep-sea sediments, they dominate the bacterial community [17], reflecting their critical roles in certain biogeochemical cycles of both organic and inorganic compounds [18]. *Shewanella piezotolerans* WP3 (hereafter called WP3) is a bacterium isolated from a western Pacific Ocean sediment sample located at a water column depth of 1,914 m [19], [20]. It is psychrotolerant and piezotolerant, growing optimally at 15–20°C and with a broad pressure optimum extending from atmospheric pressure to about 20 MPa. Because of its relatively rapid growth and broad range of physical adaptations, it provides a particularly good model for understanding *Shewanella* adaptation to the deep ocean. Here, we present the results of whole genome sequence and related functional analysis of WP3. In addition, we compared the WP3 genome with other *Shewanella* genomes, in particular with *S. oneidensis* MR-1. This is the first detailed comparative genomic analysis of *Shewanella* species inhabiting dramatically different environments (shallow lake versus deep sea).

## Results and Discussion

### General genome features

**Figure 1:**
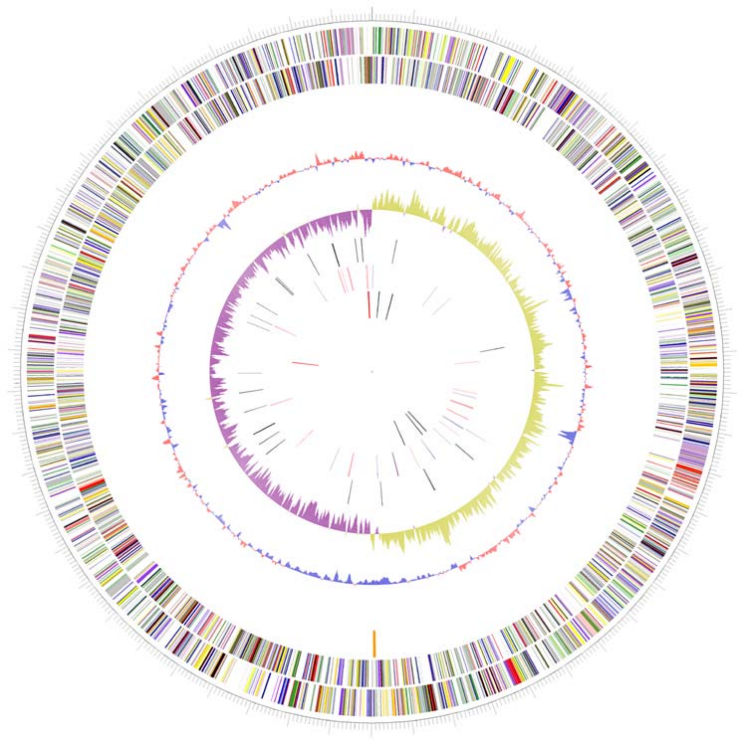
Circular representation of the *Shewanella piezotolerans* WP3 genome. From the outside inward: the first and second circles show predicted protein-coding regions on the plus and minus strands, by biological role: translation/ribosome structure/biogenesis, pink; transcription, olive drab; DNA replication/recombination/repair, forest green; cell division/chromosome partitioning, light blue; posttranslational modification/protein turnover/chaperones, purple; cell envelop biogenesis, red; cell motility/secretion, plum; inorganic ion transport/metabolism, dark sea green; signal transduction mechanisms, medium purple; energy production/conversion, dark olive green; carbohydrate transport/metabolisms, gold; amino acid transport/metabolism, yellow; nucleotide transport/metabolism, orange; coenzyme transport/metabolism, tan; lipid transport/metabolism, salmon; secondary metabolites biosynthesis/transport/catabolism, light green; defense mechanism, black; general function prediction only, dark blue; function unknown and hypothetical protein, gray. The third circle shows G+C content percentage in relation to the mean G+C% for the chromosome. The fourth circle shows GC skew. The fifth circle shows insertion sequences, retrotransposons (black) and prophage (orange). The sixth and seventh circles show tRNA genes and rRNA operons, with blue or black on the plus strand and red on the minus one.

The WP3 genome consists of a single circular chromosome of 5,396,476 bp with 4,944 predicted genes (Fig. 1). WP3 has the largest genome size among the sequenced *Shewanella* genomes. General features of the WP3 genomes are presented in Table 1. Of these predicted proteins, 3,326 (67.3%) are similar to known proteins in current databases, 877 (17.7%) are conserved hypothetical proteins, and 741 (15.0%) are hypothetical proteins which have no database match (see Supplementary Table S1, especially the 554 hypothetical proteins of 30–100 amino-acid-in-length). The genome of WP3 is mostly related to *S. loihica* PV-4 genome (supplementary Tabel S2), a psychrotolerant bacterium isolated from iron-rich microbial mats at an active, deep-sea, hydrothermal Naha vent (1,325 m below sea level) located on the South Rift of Loihi Seamount, Hawaii [21], [22]. WP3 and PV-4 have extensive regions of similar gene order (synteny), as revealed by an obvious “X” pattern when the two genomes are aligned (figure not shown); this pattern indicates that *Shewanella* genomes have undergone extensive inversions around the origin and terminus, as has been seen in other closely related bacteria [23].

**Table 1:** General features of two *Shewanella* genomes.

Genome feature	*S. piezotolerans* WP3 chromosome	*S. oneidensis* MR-1	
		chromosome	plasmid
Size, bases	5,396,476	4,969,803	161,613
G + C content, %	43.2	46	43.7
Number of predicted ORFs	4,944	4,324	148
Average ORF length, bases	926	959	780
Coding density, %	84.4	83.4	71.5
Genes with orthologue in each other	3,167	3,016	32
Genes without orthologue in each other	1,777	1,308	116
rRNA operons	8	9	0
16S-23S-5S	7	9	0
16S-23S-5S-5S	1	0	0
Number of tRNAs	89	102	0

### Proteome phylogenetic analysis

**Figure 2:**
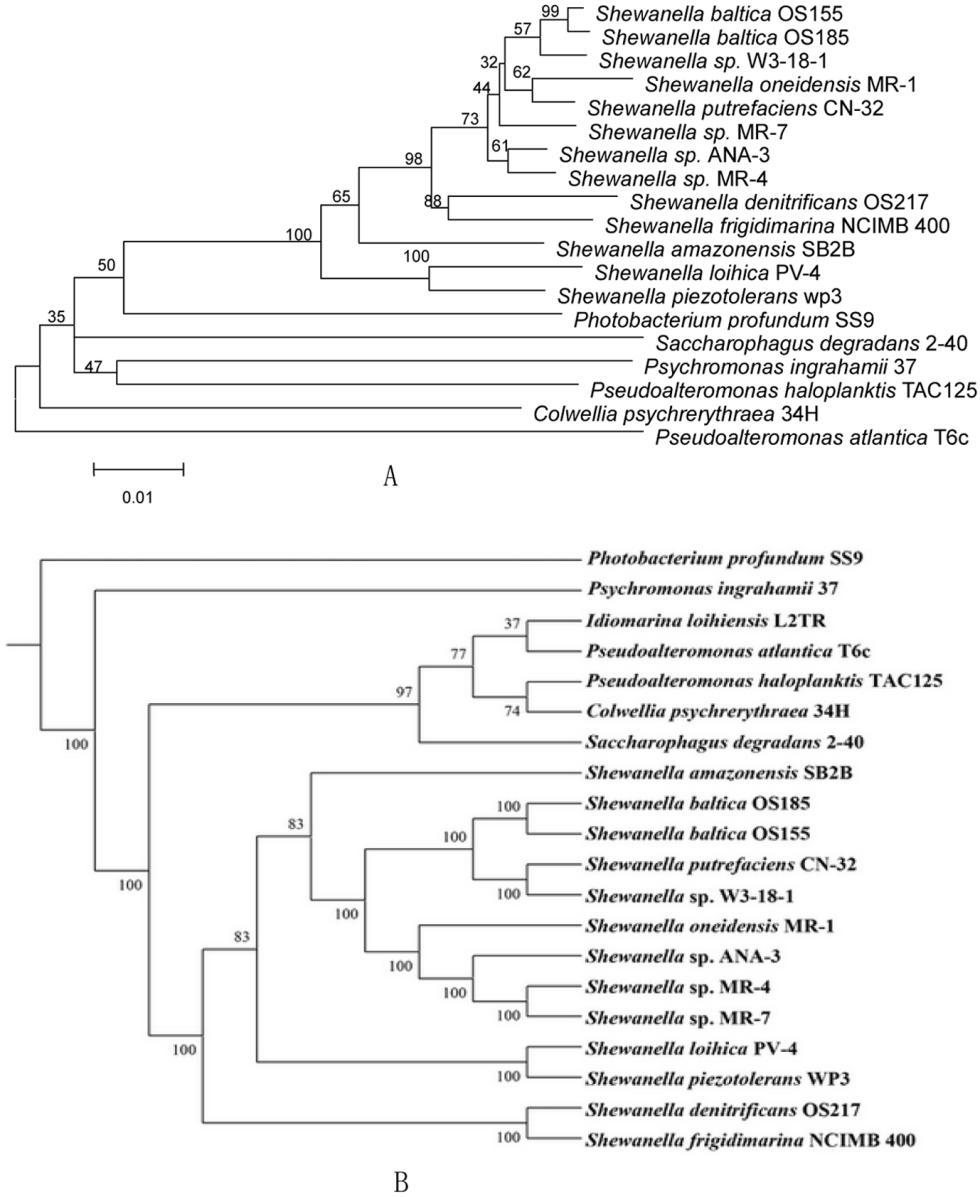
Phylogenetic tree based on 16S rRNA (A) and whole proteome analysis (B). The phylogenetic tree of the 16S rRNA gene sequences from various *Shewnaella* species was constructed by neighbor-joining method using the programs of MEGA package. 1000 trial of bootstrap analysis was used to provide confident estimates for phylogenetic tree topologies. The phylogenetic tree based on the whole proteome sequences of the strains was constructed as described in the materials and methods with 100 trial of bootstrap.

We used a whole proteome phylogenetic analysis method which utilizes the oligopeptide content, i.e., frequency of amino acid K strings in the complete proteomes to infer the evolutionary relatedness of *Shewanella* species [24]. Our analysis includes 13 sequenced *Shewanella*: *S. baltica* OS185, OS155; *S. putrefaciens* CN-32; *S. sp.* W3-18-1; *S. amazonensis* SB2B; *S. oneidensis* MR-1; *S.* sp. ANA-3; MR-4; MR-7; *S. loihica* PV-4; *S. piezotolerans* WP3; *S. denitrificiens* OS217; *S. frigidimarina* NCIMB400. The whole proteome phylogenetic tree is similar, but not entirely consistent with the 16S rRNA gene phylogenetic tree, which is widely used for species phylogenetic relationship analysis (Fig. 2A, B). Both of the proteome and 16S rRNA gene phylogenetic analysis showed that strain WP3 and PV-4, OS217 and NCIMB400 are closest related, respectively. In the proteome tree, MR-1, ANA-3, MR-4, and MR-7 formed a cluster, while strain OS185, OS155, CN-32, W3-18-1 formed another cluster. On the other hand, there is no clear cluster seperation formed by these eight strains in the 16S rRNA gene tree. Both analyses indicate that *S. oneidensis* MR-1, which has been subjected to a detail genomic and proteomic investigation, evolved after the species PV4 and WP3 during the *Shewanella* genus evolution.

### Functional classification and genome comparison

**Figure 3:**
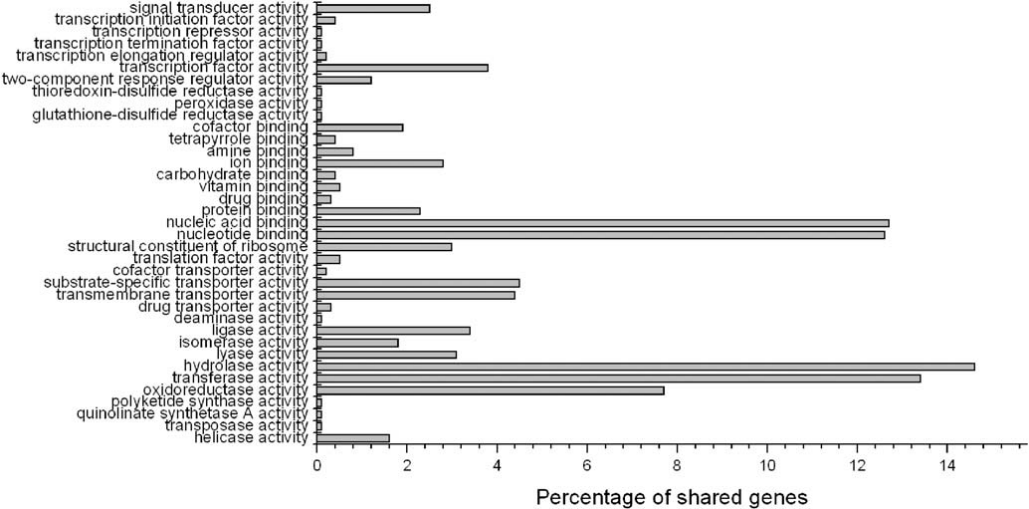
Percentage of shared genes in *Shewanella*. The function classification of shared genes in 13 compared *Shewanella genomes* was based on Gene Ontology term. Of all common genes, those belong to nucleic acid binding, nucleotide binding, hydrolase activity, transferase activity and oxidoreductase activity are predominant.

As *S. oneidensis* MR-1 has been used for detailed genomic and proteomic investigations, the COG (Clusters of Orthologous Groups) [25] functional classification of WP3 was compared with that of MR-1. The data revealed that the percentage of genes involved in most functional categories in the two genomes are quite similar (P = 0.6815). WP3 has slight increase in genes involved in cell wall/membrane/envelope biogenesis (M), energy production and conversion (C), intracellular trafficking , secretion, and vesicular transport (U) and inorganic ion transport and metabolism (P), while *S. oneidensis* has more genes in replication, recombination and repair (L), cell cycle control, cell division, chromosome partitioning (D), signal transduction (T), posttranslational modification, protein turnover, chaperones (O), nucleotide transport and metabolism (F) and coenzyme transport and metabolism (H). There are many more unclassified genes in WP3 (1823, 36.9%) than *S. oneidensis* (1365, 30.5%). Comparing the 13 complete *Shewanella* genome sequences mentioned above, 1,591 common genes were found. These common genes are supposed to constitute the core genome of the *Shewanella* genus. Of all the shared genes, most belong to nucleic acid binding, nucleotide binding, hydrolase activity, transferase activity and oxidoreductase activity based on Gene Ontology term (Fig. 3).

### Duplicated genes

Compared with other sequenced *Shewanella* genomes, the WP3 genome is the largest. We compared paralogous gene families (transposases excluded) in MR-1 and WP3 genomes. The results clearly indicated that to each pairs of corresponding gene families (paralog) in both genomes, WP3 usually has much more gene number in these families than MR-1has: WP3 contains 225 paralogous families which have more gene members (432 more genes) than their ortholog families in MR-1, while only 102 families of larger gene content (165 more genes) are present in MR-1. Supplementary Table S3 lists the expanded paralogous families in both genomes (having >2 more genes in one paralogous gene family compared to the other and >20% more genes of the average family member size; 31 versus 5 families are presented). In WP3, these expanded families are primarily involved in transport, secretion, energy metabolism and transcriptional regulation. The existence of additional duplicated genes could provide more capacity for coping with environmental change and less selective pressure because of their redundancy in WP3.

### Electron transport

**Figure 4:**
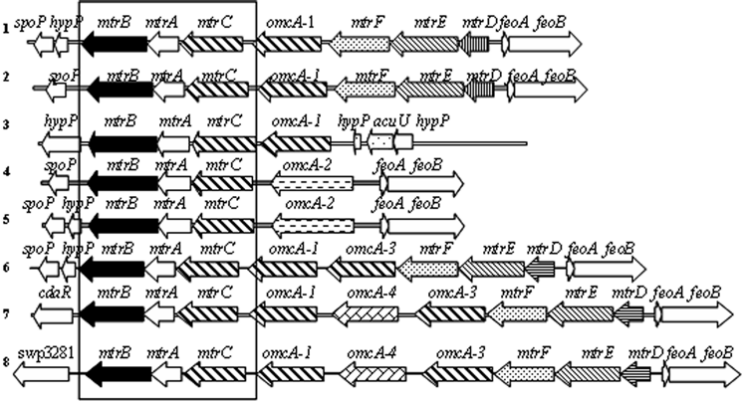
The *mtr-omc* gene cluster and its surrounding genes. The *mtr-omc* gene clusters in several *Shewanella* genomes are illustrated. The conserved *mtrBAC* operon is boxed. *hypP* represents gene for hypothetical protein, *spoP* encodes for phage SPO1 DNA polymerase domain protein. 1, *Shewanella* sp. MR4; *S.* sp.ANA3; *S*. sp. MR7; *S. baltica* OS155; 2, *S. oneidensis* MR-1; *S. baltica* OS185; 3, *S.frigidimarina* NCIMB400; 4, *S.putrefaciens* CN32; 5, *S.* sp. W3-18-1; 6, *S. amazonensis* SB2B; 7, *S. Loihica* PV4; 8, *S.piezotolerans* WP3.

*S. oneidensis* contains 42 putative cytochrome *c* genes which encode *c*-type cytochromes as constituents of its diversified respiratory network [11]. WP3 also can use a variety of electron acceptors such as nitrate, fumarate, trimethylamine N-oxide (TMAO), dimethyl sulfoxide (DMSO), and insoluble metals during anaerobic growth [20]. By careful pattern searching and BLAST analyses [11], the cytochrome *c* genes in WP3 genome were identified as listed in Table S4. Its genome contains 55 putative *c*-type cytochrome genes including 1 with 11 heme-binding sites, 11 with 10 heme-binding sites, 5 with 7 or 8 sites, and 12 with 4 or 5 sites. According to sequence similarities, the cytochromes *c* genes in WP3 could be divided into 17 groups as shown in Supplementary Table S5. WP3 contains many more decaheme cytochome *c* and nitrite reductase genes than MR-1 (17 and 5 versus 10 and 1, respectively), while it has less fumarate reductase genes than MR-1. Among the sequenced *Shewanella*, only the *S. denitrificiens* OS217 does not appear to be able to reduce metals. There are 10 hypothetical outer membrane lipoproteins in WP3 which are probably involved in the insoluble substance reduction. The *mtrABC*/*omcA* gene cluster which is known in MR1 to participate in the metal reduction is conserved in all the *Shewanella* genomes except OS217. Fig. 4 shows the *mtrCAB* operon and its upstream and downstram genes in the *Shewanella* genomes. Upstream the *mtrBAC* operon, an *omcA* or hypothetical *omcA* like genes follow. Four types of *omcA* genes, here named as *omcA-1* to *omcA-4,* were identified. Strains CN32 and W3-18-1 contain an 11 heme-binding cytochrome *c* gene-*omcA2*, and both of these two strains together with NCIMB400 lack the *mtrDEF* which are conserved in some of the *Shewanella* genomes. WP3 also contains an 11 heme-binding *cytC* gene-swp3899 which is not involved in the *mtr*-*omc* gene cluster. The protein OmcA and MtrC are known to form a complex to reduce metal oxides *in vitro*. Upstream the *mtrC* gene, strain SB2B contains two hypothetical *omcA* genes, while strain PV4 and WP3 contain 3 hypothetical *omcA* like genes. Most of the gene cluster is preceded by a ferrous iron transporter gene cluster *feoAB* which is only absent in NCIMB400. Downstream the *mtrCAB* operon, there exists a phage SPO1 DNA polymerase domain protein (named as *spoP*) in most of the genomes. Reports have suggested that the membrane-based cytochrome respiratory system may not be fully functional at high pressure, and the expression of genes for some respiration components is regulated by hydrostatic pressure [5], [26]. We are making *cytC* gene mutations to investigate the functions of the CytCs in WP3 living under different environments. The high number of cytochrome *c* genes in WP3 could be an adaptation to high pressure deep-sea environments.

### Metabolic pathways

Compared to MR-1, WP3 has a great number of genes involved in various metabolic pathways (Supplementary Table S6). These include genes for the catabolism of starch and sucrose, pentose, fructose and mannose, ascorbate and aldarate, galactose, nucleotide sugars and aminosugars; glycan metabolism (e.g., glycosphingolipid and glycosaminoglycan); amino acid metabolism (e.g., arginine, proline and beta-alanine) amine and urea cycle metabolism; and the breakdown of assorted other compounds such as glycerolipids, porphyrins and chlorophyll. Besides these enzymes, there are also genes encoding specific transporters, transferases and regulators involved in the above pathways (Supplementary Table S7). WP3 inhabits an oligotrophic, cold and high pressure deep-sea environment. The existence of so many carbon and energy utilization pathways and osmolyte transport and synthesis systems is a reflection of the adaptation to this setting. Likewise, the absence of any light-activated photolyase genes indicates an existence free of ultraviolet light (in contrast *S. oneidenis* contains one deoxyribodipyrimidine photolyase gene). This is consistent with the absence of sunlight in the deep-sea environment WP3 inhabits.

WP3 contains eight putative sulfatase genes, whereas *S. oneidensis* contains only one (SO4628, sulfatase). Bacterial sulfatases are known to function primarily in sulfur scavenging. Since marine systems are characterized by high inorganic sulfur concentrations, the limitation of this element would be expected to be rare where WP3 is found. However, sulfatases have been observed in other marine bacteria. The marine bacterium *Pirellula* sp. strain 1 harbors 110 putative sulfatase genes in its genome, which is the largest number known at the present time [27]. It has been suggested that *Pirellula* sp. strain 1 uses the sulfatases to efficiently degrade sulfated glycopolymers to gain energy rather than to meet its sulfate requirements. The sulfatase genes in WP3 may have a similar function. In addition, there are three other WP3 specific genes, with four copies each, involved in sulfur metabolism (Supplementary Table S8). These appear to be sodium/sulphate symporters (COG0471), arylsulfatase regulators (Fe-S oxidoreductase, COG0641), and glutathione S- transferases (COG0625).

### WP3 Membrane Fluidity

**Figure 5:**
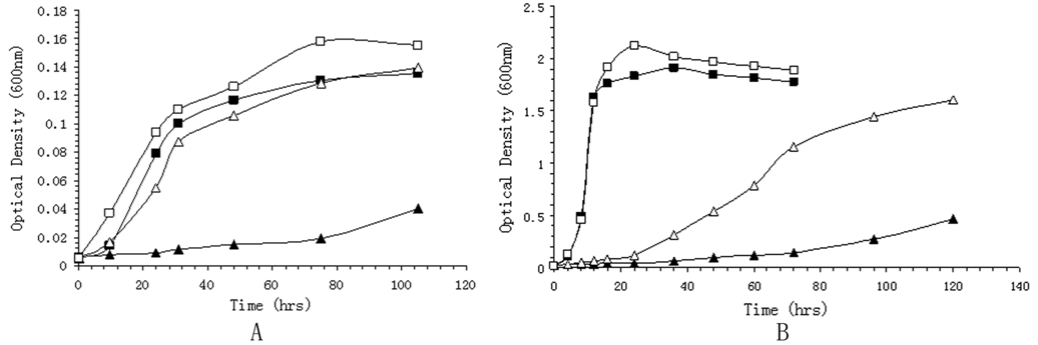
Growth curve of WP3 and its mutant. The growth of WP3 and the WP3Δorf2 mutant under different temperature and pressure conditions were monitered by measuring the optical density of the cultutes at 0D_600_. For each measurement, triplicate cultures were measured simultaneously, and the average value was recorded. A: Growth of the strains at 0.1 MPa and 20 MPa. Wild type WP3 was grown at 0.1 MPa (□), or at 20 MPa (▪); WP3Δorf2 mutant grown at 0.1 MPa (▴) or 20 MPa(Δ); B: Growth of the strains at 4°C and 20°C. Wild type WP3 was grown at 4°C (□), or at 20°C (▪); WP3Δorf2 mutant grown at 4°C (▴) or 20°C (Δ).

A change in membrane fluidity is a well-established response of poikilothermic organisms to low temperature and high pressure exposure [3]. WP3 can synthesize eicosapentaenoic acid (EPA; 20:5), and its content in the cell membrane increases as a function of the decreased temperatures [19]. WP3 harbors a gene cluster (WP3546–3554) for eicosapentaenoic acid (EPA; 20:5) omega-3 polyunsaturated fatty acid synthesis. Five genes in this gene cluster have been shown essential for EPA synthesis [28]. Mutation of the orf2 gene which encodes the putative phosphopantetheinyl transferase (PPTase) diminished the EPA synthesizing capability of WP3, and the Δorf2 WP3 strain showed much slower growth rate under low temperature or high pressure compared with the wild type strain (Fig. 5). On the other hand, in the deep-sea bacterium *Photobacterium profundum* SS9, it has been observed that EPA is not essential for the growth of SS9 at low temperature and high pressure. We are still not clear why EPA plays different roles in the environmental adaptation of WP3 and SS9. Comparison of the genome of WP3 with those of PV-4, *Desulfotalea psychrophila* Lsv54, *Colwellia psychrerythraea* 34H and *Photobacterium profundum* SS9, all of which are cold-adapted bacteria, only indicated the presence of a few features in common: WP3, PV-4, 34H and SS9 all contain the EPA synthesis gene cluster, and Lsv54 contains two copies of β-keto-acyl-CoA synthase I, which elongates unsaturated fatty acids. This further illustrates that maintaining membrane physical structure is crucial for life in cold and/or high pressure environments, and this can be achieved by increasing the ratio of unsaturated fatty acids.

### tRNA, rRNA modifications

WP3 contains seven pseudouridine synthase genes (COG1187), nine pseudouridylate synthase genes (COG0564) and three genes (COG0101, COG0130, COG4445) for RNA modification [29], [30] (Supplementary Table S9). In general, bacteria only have 1–4 copies of these genes; this is the first report of so many pseudouridine synthase genes in a single bacteria genome. The high number of pseudouridine synthase genes was also observed in several of the *Shewanella* genomes including PV4, MR-4, MR-7, CN-32, SB2B, OS155, 0S217, but absent in the MR1 genome. Khachane *et al* reported that the uracil content of 16S rRNA in thermophilic and psychrophilic prokaryotes increase as their optimal growth temperatures drop [31]. Previous studies have also indicated that the maintenance of flexibility at low temperatures is important for tRNA function, and this is mainly achieved through post-transcriptional incorporation of dihydrouridine in psychrophilic bacteria [32]. Cellular RNA contains a number of post-transcriptionally modified nucleosides, the most common of which is pseudouridine (ψ). Pseudouridine has been identified in tRNAs, rRNAs, snRNAs and/or snoRNAs for which the stability of their tertiary structure is important for function [29], [33]. Pseudouridine synthases are the enzymes responsible for formation of the ψ residues. In *E. coli*, the pseudouridine synthase RluD is responsible for the formation of ψ1911, ψ1915, and ψ1917 in 23S rRNA. Disruption of the *rluD* gene and/or loss of the pseudouridine residues for which it is responsible resulted in a severe growth phenotype. RluD and/or the ψ1911, ψ1915, and ψ1917 residues formed by it are necessary for normal ribosome biogenesis, assembly, and function in *E. coli* [29], [34]. The large number of genes for structural RNA modification in so many *Shewanella* genomes may be one of the important mechanisms for the wide environmental adaptation and distribution of the genus.

### Cell motility

**Figure 6:**
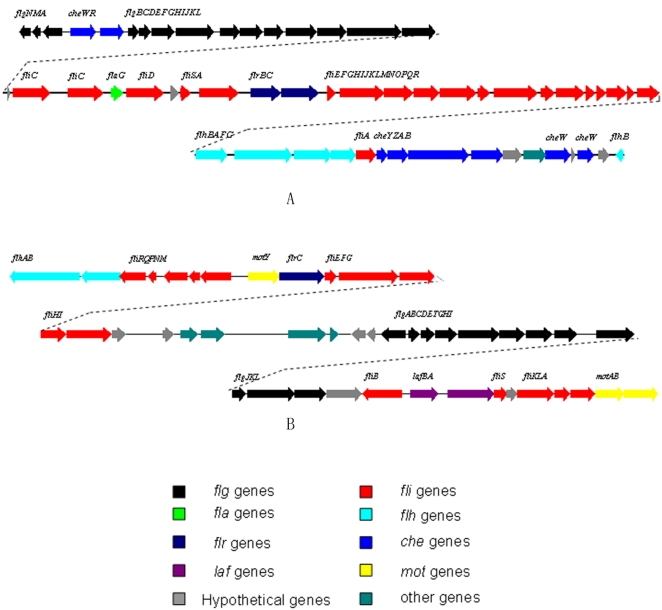
Gene organization map of two flagellum systems in WP3. A: Flagellum gene cluster I (polar): swp1493—swp1550; B: Flagellum gene cluster II (lateral): swp5082—swp5126. Arrow indicates gene transcription direction.

**Figure 7:**
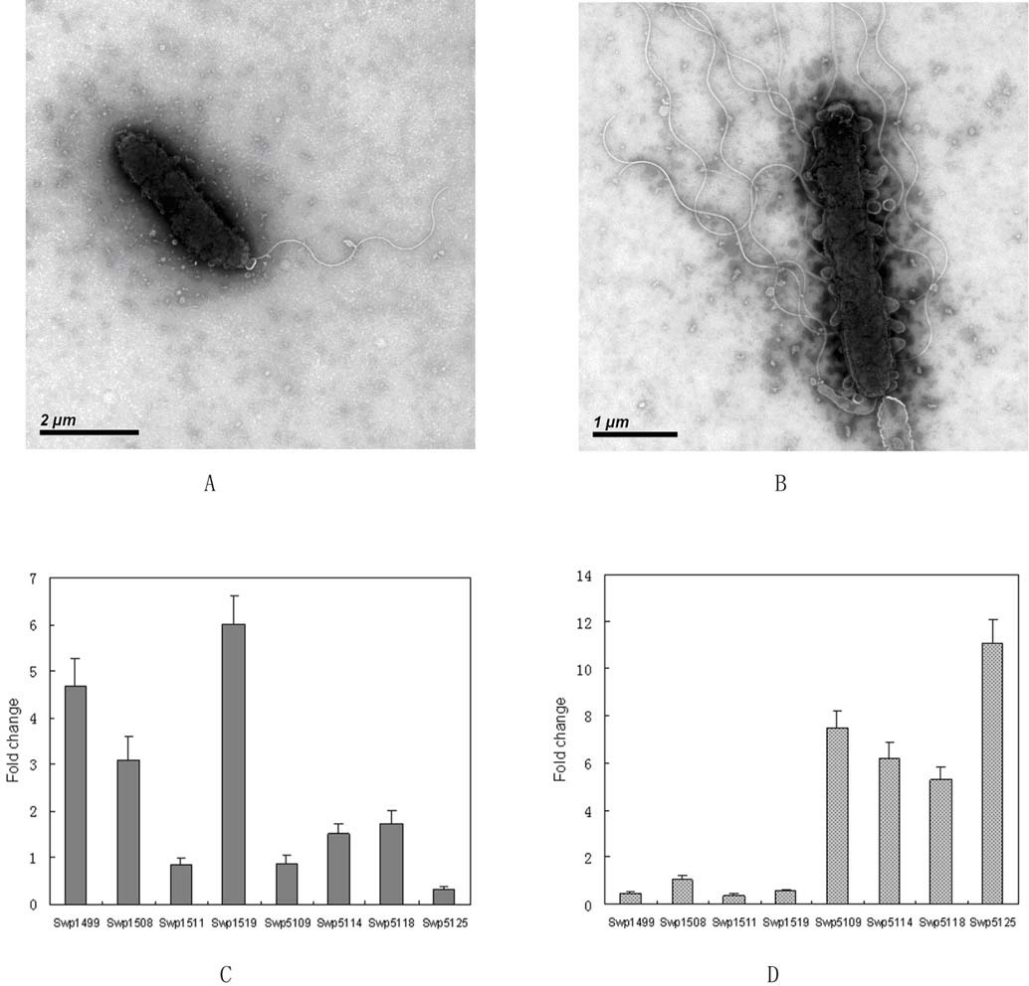
TEM image of polar (A), lateral ( B) flagella and the transcriptional regulation of genes in flagellum synthesis under different pressures (C) and temperatures (D). The WP3 cells from liquid culture and from agar plates were visualized by TEM separately. The cells from liquid culture were shown to have polar flagellum (A); while the cells from ager plates also contain lateral flagellum (B). The transcription level of four genes (swp1499, swp1511, swp1508, swp1519) for polar flagellum synthesis and four genes (swp5109, swp5114, swp5118, swp5125) for lateral flagellum synthesis under different temperatures and pressures were checked by Quantitative RT-PCR as described in the materials and methods. C: The transcription changes of genes at 20 MPa as compared with that at 0.1 MPa. The transcription levels of the genes at 0.1 MPa are set as control; D: The transcription change of genes at 4°C as compared with that at 20°C. The transcription levels of the genes at 20°C are set as control

**Figure 8:**
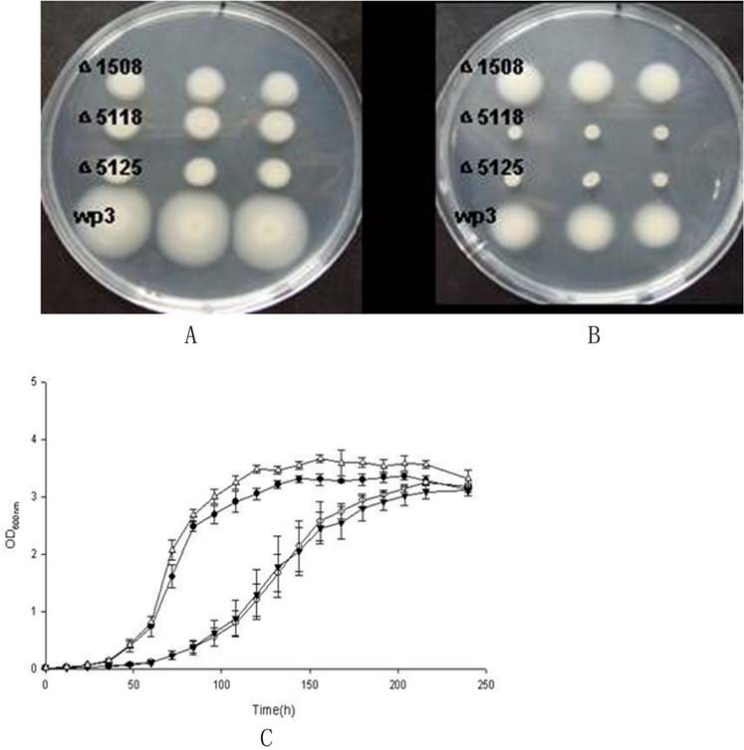
Motility and growth assays of the WP3 strains. The motility assays for WP3 and the mutants (WP3Δ1508 has defect in polar flagellum; WP3Δ5118, WP3Δ5125 defect in lateral flagellum) were conducted on 0.3% agar plates at 20°C (A) or 4°C (B). Triplicates of each strain were spotted onto the soft agar, the motile ability of the strains were compared by observing the movements of the strains after 3 days at 20°C, or 7 days at 4°C. The growth of WP3 and the mutants at 4°C was monitered by measuring the optical density of the cultutes at 0D_600_. The mean values with standard deviations (indicated by vertical bars) from triplicate experiments are given. Δ represents the wild type WP3; • mutant WP3Δ1508; ○WP3Δ5118 ▾WP3Δ5125.

Two sets of flagella genes are present in the WP3 genome (flagella I: swp1493-swp1550, flagella II: swp5082-swp5126, Fig. 6). Flagella I contains the *flgM, flgN, fliJ* and *fliO* genes, which are missing in the flagella II gene cluster. Two stator proteins, MotY, MotAB (swp5089, swp5126,5127), responsible for converting proton motive force into torque against the rotor/switch, are found to be encoded in the flagella II gene cluster. No motor protein genes were found near the Fla I gene cluster area. However, two clustered genes, swp3615 and swp3616, which code for putative MotB and MotA proteins are found in the genome far away from both of the flagella gene clusters. These two genes could be responsible for the rotor/switch function of Fla I or both. Sequence comparison results suggest that the flagella I and II in WP3 correspond to the polar and lateral flagella of *V. parahaemolyticus* [35]. The polar flagellum propels the bacterium in a liquid environment (swimming), while the lateral flagella are responsible for movement over surfaces or viscous environments (swarming) [36]. The WP3 cells in the liquid cultures only contains a single polar flagellum as observed by transmission electronic microscope (Fig. 7A), while the lateral flagella was induced when cultivated on agar plates (Fig. 7B). The two flagella systems could facilitate the movement of WP3 cells in sediment pore fluids or in seawater from planktonic to surface-associated lifestyles. Interestingly, the two sets of flagellum systems were found to be inversely regulated by low temperature and high pressure (our unpublished microarray data). The differential regulation of the flagellum by temperature and pressure was verified by quantitative RT-PCR (Fig. 7C, D). As shown in the figure, the genes of lateral flagellum were highly up regulated by low temperature (4°C versus 20°C) and slightly repressed by high pressure (20 MPa versus 0.1 MPa); while the genes of polar flagellum were largely repressed at low temperature and highly activated at high pressure. Mutants which have defects in polar or lateral flagella synthesis (WP3Δ1508 defect in polar flagellum; WP3Δ5118, WP3Δ5125 defect in lateral flagellum) showed decreased motility on 0.3% agar plates at 20°C (Fig. 8A). The mutant lacking lateral flagella completely lost motility at 4°C, while the mutant with a defect in the polar flagellum remained motile (Fig. 8B). This clearly indicated that the lateral flagellum is essential for the motility of the strain at low temperature. Strains WP3Δ5118 and WP3Δ5125 showed decreased growth rate compared to the wild type when grown at 4°C (Fig. 8C), while no growth arrest was observed at 20°C. This is the first demonstration of a requirement for a second flagellum system for growth at low temperature. The deep-sea bacterium *Photobacterium profundum* strain SS9 also has two sets of flagella genes [4] and thus the results presented here may extend to multiple genera of deep-sea microbes. The nature of the connection between swimming speed and anabolism and cell division are unknown and will require further investigation.

### WP3 Phage

The WP3 genome includes only one prophage, a small 7,718 bp filamentous phage genome (named SW1), while MR-1 contains three prophages including one lambda-like phage (LambdaSo, 51,857 bp) and two phylogenetically distinct phages related to the *E. coli* Mu (MuSo1, 34,551 bp and MuSo2, 35,666 bp). Phage SW1 is integrated into the terminus of the chromosome and also exits as an extra-chromosomal plasmid. It contains nine putative open reading frames (ORF) as listed in Table 2. This is the first filamentous phage found in a *Shewanella* species and could be a useful genetic engineering tool. Filamentous phages are unlike most other bacterial viruses in that the infected host remains viable and phages are continuously assembled and extruded from the cell in a concerted process [37]. Most of the sequences of the SW1 phage ORFs share low but significant similarities with those of *Vibrio* filamentous phages. SW1 could be assigned to genus *Inovirus* of the family *Inoviridae* of bacteriophages. The SW1 prophage is flanked by 13 base pair (GAATGCGCACAAT) direct repeats. Viable filamentous phage was successfully isolated from WP3 and it was found significantly induced and released at low temperature [38]. WP3 lives in the permanent cold deep-sea environments, at which condition the filamentous phage is highly induced and released into the environments. The significance of this character for the species environmental adaptation and evolution is still unclear. Similarity searches among sequenced *Shewanella* genomes indicated that two strains, *Shewanella baltica* OS155 and *Shewanella putrifaciens* CN-32, contain sequences similar to those of filamentous phage. These two DNA fragments are adjacent to the phage integrase gene, suggesting that they are derived through phage mediated horizontal gene transfer. The roles of filamentous phage in *Shewanella* evolution await further clarification.

**Table 2:** Putative ORFs in the SW1 phage sequence and the BLAST search results.

ORF of SW1*	Homologous gene products from phage or bacterium			Identity %	Accession number
	Gene product	Phage	Host bacterium		
ORF541	RstA-like protein	CTX phi	*Vibrio cholera*	52%	ABC47903
ORF104	RstB like Protein	CTX phi	*Vibrio cholera*	50%	ABC47904
ORF44	Putative major capside Protein	VGJ phi	*Vibrio cholerae*	59%	NP_835475
	Hypothetical Protein fs1P01	fs1	*Vibrio Cholerae*	59%	NP_695190
ORF61	Hypothetical protein		*Vibrio splendidus*	51%	ZP_00991919
ORF79	Phage-related protein		*Xylellea fastidiosa*	50%	AAO28802
ORF527	Hypothetical protein		*Shewanella baltica*	37%	ZP_00583158
	Putative adsorption Protein	VGJ phi	*Vibrio Cholerae*	32%	AAO93101
ORF105	Vpf104	Vf33,12	*Vibrio parahaemolyticus*	37%	YP_031684
ORF382	Putative Zonular occudens toxin		*Shewanella baltica*	62%	ZP_00583157
	Putative assembly protein	VGJ phi	*Vibrio cholerae*	35%	NP_835479
ORF116	Transcription regulator		*Lactobacillus casei*	31%	YP_807254

* The gene names are designated by the number of amino acid residues they contain.

### Conclusion

The complete genome sequence of the deep-sea bacterium *Shewanella piezotolerans* WP3 reflects a capacity for coping with diverse energy sources and variable physical conditions. The WP3 genome contains genes or gene clusters which enhance its capacity for nutrient acquisition, energy production, macromolecule synthesis and protein function under deep-sea conditions. These include genes for two sets of flagellum systems, structural RNA modification, functional EPA synthesis, and osmolyte synthesis and transport. WP3 contains 55 putative cytochrome c genes, indicating a versatile respiratory adaptation strategy and bioenergetic modification of electron flow. Understanding how different *Shewanella* species sense and respond to diverse physiochemical parameters holds the promise of extending the environmental range of their biotechnological applications.

## Methods

### Bacterial cultivation

*Shewanella piezotolerans* WP3 was isolated from a shallow sediment sample obtained from the western Pacific Ocean (E142°30′08″, N8°00′11″) at a water column depth of 1914 m. It was grown in modified Marine 2216E broth at 20°C [20]. For high pressure incubation of WP3, overnight log phase atmospheric pressure cultures of WP3 cells were grown and then diluted 1000-fold by the same medium, transferred into sterile injection syringes and placed inside pressure vessels. Cultures were then incubated at 20°C at either atmospheric pressure or at 20 MPa with the same method as described elsewhere [39].

### Sequencing and assembly

DNA isolation, cloning, sequencing, and assay were performed as described for other genomes sequenced by Beijing Institute of Genomics/Hangzhou Genomics Institute ( BIG/HGI) [40], [41]. We constructed three pUC18 plasmid libraries, with insertion size of 2K, 3K, and 5K respectively. Sequences were generated by using ABI3730 and MegaBase 1000 automatic sequencers and assembled by Phred/Phrap/Consed software [42], [43], [44]. Scaffolds were established by contigs, according to linking information from forward and reverse sequence ends of each clone. Sequence gaps were closed by re-sequencing the end sequences of each contig, primer walking on linking clones, and by sequencing PCR products from genomic DNA. Consensus sequence coverage and base ambiguities were improved by re-sequencing existing DNA samples and by sequencing additional PCR products, together with manual editing. All repeated regions were verified with PCR amplification across the repeat and sequencing the product. The final genome is based on 85,529 reads, with a total coverage of 6.52X (Q20) and overall base error probability 0.26/10 Kb.

### Genome analysis

The replication origin was determined by sequence similarity to the origin of *Shewanella oneidensis* and GC nucleotide skew analysis [45]. An initial set of open reading frames (ORFs) were predicted by Glimmer software [46]. Both predicted ORFs and intergenic sequences were subjected for further manual inspections. All predicted proteins >30 amino acids were searched against GenBank non-redundant protein database using BLAST program [47]. Frameshifts and point mutations were detected and corrected where appropriate. Remaining frameshifts and point mutations are considered to be authentic and were annotated as “authentic frameshift” or “authentic point mutation”, or, in the case of multiple lesions within a single ORF, “degenerate”. tRNAs were predicted by tRNAscan-SE [48].

The predicted proteins were further investigated by InterProScan program to InterPro database [49], which now included 14 integrated databases of protein families, domains and functional sites. Functional classification of WP3 proteins were done according to COG functional category [25]. Metabolic pathways were analysis with KEGG database [50].

### Proteome phylogenetic analysis

The whole proteome phylogenetic analysis of the speices were conducted by the Composition Vector method as described by Qi *et al.* [24]. First, we collected all amino acid sequences of a species, and then calculated the frequency of appearance of overlapping oligopeptides of length K. A random background needed to be subtracted from these frequencies by using a Markov model of order (K−2) in order to diminish the influence of random neutral mutations at the molecular level and to highlight the shaping role of selective evolution. Then, we put the "normalized" frequencies in a fixed order; a composition vector of dimension 20K was obtained for each species. And the correlation C (A, B) between two species A and B was determined by taking projection of one normalized vector on another, i.e., taking the cosine of the angle between them. Finally, the normalized distance between the two species was defined to be D = (1−C)/2. Once a distance matrix has been calculated, we then constructed phylogenetic trees by following the standard neighbor-joining method [51] in the Phylip package.

### Bacterial cultivation for RNA isolation

WP3 was cultivated as introduced above. As for different temperature conditions, overnight log phase cultures of WP3 cells diluted 1000-fold by the same medium were grown at 4°C or 25°C for 3 days to reach an equivalent optical density. All samples were in triplicate for convenience of reproductivity. The cells were immediately centrifuged and RNA was extracted using TRI reagent-RNA/DNA/protein isolation kit (Molecular research center, Inc., Cincinnati, USA). The RNA was stored in 75% ethanol at −80°C

### Transmission Electron microscopy and swimming motility assay

Strains were grown to mid-log phase in 2216E medium, centrifuged(5000 g, 1 min), and resuspended in distill water. Samples were placed to a carbon-coated grid (200 mesh) and stained with 1% phosphotungstic acid (PTA). The grid was air dried and examined in a JEM-1230 microscope (JEOL, Tokyo, Japan). The swimming motility of the strains was assessed by examining their movements in the 0.3% 2216E agar plates. The assays were initiated by spotting 1 µl of an overnight cell culture which has been normalized to an optical density of around 1.5 at OD _600nm_ onto the agar plates, then incubated at 20°C or 4°C for 3 days or a week, respectively.

### Real Time RT-PCR

Three genes for polar and four genes for lateral flagellum synthesis were used for transcription analysis using the Real time RT-PCR method. The primer pairs of selected genes for real-time PCR were designed using Primer Express software (ABI). The primer sequences are: swp5109 For 5′-CCCGCTCAGGT GCCATAG, swp5109Rev 5′-CAA TAGG GCGGCCATCTAAG; swp5114 For 5′-ACCTGTTTAGCTACGATCCCAGTA, swp5114Rev 5′-AGCCAACATATCA GCGTTGAAG; swp5118 For 5′-GCAGATGGTGCATTAGACGAACT, swp5118Rev 5′-TTCACGCCGTTAGCTGCTT; swp1499 For 5′-GCAGCGCAGGCGAAACT, swp1499Rev 5′-TGCTGTTGTGCTTTG CTCATT; swp1511 For 5′-GCACAAAGC GCGATTGC, swp1511Rev 5′-CGGTTCTGTACCGCACCTAAG; swp1508 For 5′-G GGTGGACTTGATGTCAATGTG, swp1508 Rev 5′-TTAGAGGTTGAGTCGCATCAAAAT; swp1519 For 5′-GAAATGTG AGCGAACTTCAGTCA, swp1519Rev 5′-TGTCACTGTGGTATCGCCCATA; swp5125 For 5′-GGCTGCACAACTACGATGTGTT, swp5125 Rev 5′-GGCGGTTTGCCTTTAGCA. PCR cycling was conducted using 7500 System SDS software in reaction mixtures with total volumes of 25 ul containing 1× Sybr Green I Universal PCR Master Mix (ABI), 0.5 uM each primer, 1 ul cDNA template. In this method, the amount of target was normalized to that of the reference gene swp0453 whose expression remains stable under various conditions, relative to the calibrator (The transcription levels of the genes at 0.1 MPa, or 20°C were set as 1). Real time RT-PCR assays were performed in triplicate for each sample, and a mean value and standard deviation were calculated for the relative RNA expression levels.

### Accession number

The sequence has been deposited in GenBank with accession number CP000472.
