# Correction: Environmental Adaptation: Genomic Analysis of the Piezotolerant and Psychrotolerant Deep-Sea Iron Reducing Bacterium *Shewanella piezotolerans* WP3

**DOI:** 10.1371/annotation/744d7c8d-db8a-4ad4-aec5-62549b1dbefe

**Published:** 2008-05-08

**Authors:** Fengping Wang, Jianbin Wang, Huahua Jian, Bing Zhang, Shengkang Li, Feng Wang, Xiaowei Zeng, Lei Gao, Douglas Hoyt Bartlett, Jun Yu, Songnian Hu, Xiang Xiao

The Supplementary Tables do not appear in the manuscript. They can be viewed here:
