# Supplementary material for: Correction: Environmental Adaptation: Genomic Analysis of the Piezotolerant and Psychrotolerant Deep-Sea Iron Reducing Bacterium Shewanella piezotolerans WP3
Source: PLoS One. 2008 May 8;3(5):10.1371/annotation/744d7c8d-db8a-4ad4-aec5-62549b1dbefe. doi: 10.1371/annotation/744d7c8d-db8a-4ad4-aec5-62549b1dbefe (PMC2638141; doi:10.1371/annotation/744d7c8d-db8a-4ad4-aec5-62549b1dbefe)
Supplement: Supplementary file 1 [file pone.744d7c8d-db8a-4ad4-aec5-62549b1dbefe.s001.pdf]

PLoS PUBLIC LIBRARY of SCIENCE

Table S1. Gene size and their number

Protein length	Total proteins	Functional proteins	Conserved hypothetical proteins	Hypothetical proteins
30-100	826	145	127	554
101-200	1096	643	337	116
201-300	973	743	192	38
301-400	788	661	105	22
401-500	502	446	52	4
>501	759	688	64	7

Table S2. WP3 ORF blast in the sequenced *Shewanella* genomes

Bacterium	#ORF of BLAST Hit	#ORF of Best BLAST Hit
Shewanella loihica PV-4	2757	1634
Shewanella baltica OS185	2594	262
Shewanella baltica OS155	2557	157
Shewanella sp. MR-4	2550	80
Shewanella sp. ANA-3	2547	173
Shewanella sp. MR-7	2541	110
Shewanella frigidimarina NCIMB 400	2496	283
Shewanella putrefaciens CN-32	2477	171
Shewanella sp. W3-18-1	2451	48
Shewanella oneidensis MR-1	2319	100
Shewanella amazonensis SB2B	2303	81
Shewanella denitrificans OS217	2149	110

Table S3. Gene paralog families in *S. piezotolerans* WP3 and *S. oneidensis* MR-1.

Paralogous gene family	WP3	MR-1	Differ*	Differ%**
VCBS/collagenase/Peptidase/Decaheme cytochrome c	62	40	22	41.9
HlyD family secretion protein	42	22	20	59.7
TonB-dependent receptor	41	26	15	44.8
Acriflavin resistance protein	33	18	15	57.7
Transcriptional regulator, LysR family	65	53	12	20.2
MSHA pilin protein/secretory pathway	21	13	8	47.1
Aldehyde dehydrogenase	18	12	6	38.7
Acetyltransferase, GNAT family	10	4	6	85.7
Short-chain dehydrogenase/reductase SDR	21	16	5	27
NADH:ubiquinone oxidoreductase, Na(+)-translocating, F subunit	9	5	4	57.1
NADH dehydrogenase	9	5	4	57.1
Nitrite reductase (cytochrome; ammonia-forming)	5	1	4	133
Glucose/galactose transporter	15	12	3	22.2
Dihydrolipoamide acetyltransferase	15	12	3	22.2
Aminotransferase, class V	12	9	3	28.6
AMP-dependent synthetase and ligase	11	8	3	30
MotA/TolQ/ExbB proton channel	9	6	3	40
RNA polymerase sigma-70 factor, ECF subfamily	8	5	3	46.2
TonB, C-terminal	6	3	3	66.7
MscS Mechanosensitive ion channel	6	3	3	66.7
Flagellar basal body rod protein	6	3	3	66.7
Flagellar basal body rod protein	6	3	3	66.7

MoxR protein	5	2	3	85.7
Von Willebrand factor type A domain protein	4	1	3	120
Esterase, putative	4	1	3	120
Glutathione S-transferase	4	1	3	120
Tryptophan halogenase	4	1	3	120
Sodium/sulphate symporter	4	1	3	120
Glutathione-dependent formaldehyde-activating, GFA	4	1	3	120
Conserved hypothetical protein	4	1	3	120
Conserved hypothetical protein	4	1	3	120
DTDP-glucose 4,6-dehydratase	8	11	-3***	-32
Ubiquinone/menaquinone biosynthesis methyltransferase	4	7	-3	-55
sulfate ABC transporter, permease protein	9	14	-5	-43
Tetraheme cytochrome c	8	16	-8	-67
Conserved hypothetical protein	7	19	-12	-92

* Differ: the different number of genes between WP3 and MR1 in each paralogous gene family

** Differ%: differ #/average number of family genes × 100

*** -: represent genes fewer in WP3 family than in MR-1

.

Table S4 Cytochrome *c* genes in WP3 genome

Synonym	Product	Heme binding sites	Correspondence in MR-1	Protein Identity	Putative location	Putative function
swp3899	decaheme cytochrome c MtrF	11	SO1780	28%	OM	Presumably like OmcA
swp0721	decaheme cytochrome c	10	SO1779	36%	OM	MnO2 reductase
swp0896	decaheme cytochrome c	10	SO1659	43%	OM	Presumably like OmcA
swp3272	Doubled CXXCH motif	10	SO1782	77%	Periplasmic	MtrD
swp3274	decaheme cytochrome c MtrF	10	SO1780	57%	OM	Presumably like OmcA
swp3275	decaheme cytochrome c	10	S01779	30%	OM	MnO2 reductase
swp3276	decaheme cytochrome c	10	S01779	25%	OM	MnO2 reductase
swp3277	decaheme cytochrome c	10	SO1779		OM	MnO2 reductase
swp3278	deca-heme c-type cytochrome	10	So1778	56%	OM	MnO2 reductase
swp3279	decaheme cytochrome c MtrA	10	SO1777	85%	Periplasmic	Donor to OM cyts
swp3461	Doubled CXXCH motif	10	SO1427	67%	Cyt Mem Pr	Donor to DMSO reductase
swp3466	Decaheme cytochrome c related protein	10	SO1780	23%	OM	Presumably like OmcA
swp0694	Doubled CXXCH motif	8	SO0479	22%	Periplasmic	
swp0920	OmpA-like transmembrane region:Fibronectin, type III: Putative Ig	8	SO0479	20%	Periplasmic	
swp2414	tetratricopeptide repeat family protein	8	SO1782	24%	Periplasmic	
swp4579	cytochrome c, putative	8	SO4144	72%	Cyt Mem Pr	
swp4657	cytochrome c, putative	7	SO0479	66%	periplasmic	
swp3990	NapC/NirT cytochrome c, N-terminal	5	SO1233	84%	Cyt Mem Pr	Donor to TMSO reductase
swp4117	formate-dependent nitrite reductase	5	SO4360	35%	periplasmic	Donor to DMSO reductase
swp0221	tetraheme cytochrome c	4	SO3623	49%	Cyt Mem Pr	
swp0613	putative Formate-dependent nitrite reductase,periplasmic	4	SO3980	36%	Cyt Mem Pr	Nitrite reductase

	cytochrome c552 subunit					
swp0614	Nitrite reductase (cytochrome; ammonia-forming)	4	SO3980	37%	Cyt Mem Pr	Nitrite reductase
swp0919	Nitrite reductase (cytochrome; ammonia-forming)	4	SO3980	59%	Cyt Mem Pr	Nitrite reductase
swp2772	Nitrate/TMAO reductases, membrane-bound tetraheme cytochrome c subunit	4	SO1233	48%	Cyt Mem Pr	Donor to TMAO reductase
swp2923	cytochrome c3	4	SO2727	61%	periplasmic	Electron shuttle
swp3403	Nitrite reductase (cytochrome; ammonia-forming)	4	SO3980	38%	Cyt Mem Pr	Nitrite reductase
swp3958	Nitrite reductase (cytochrome; ammonia-forming)	4	SO3980	72%	Cyt Mem Pr	Nitrite reductase
swp4352	flavocytochrome c precursor	4	SO0970	60%	periplasmic	Fumarate reductase
swp4806	NapC/NirT cytochrome c, N-terminal	4	SO4591	80%	Cyt Mem Pr	Nitrite, fumarate, Fe, Mn, reductase
swp2269	Hypothetical protein	3	SO4572	30%	Cyt Mem Pr	
swp1936	Conserved hypothetical protein	2	SO2931	56%	OM	
Swp1937	Hypothetical diheme C	2	SO2930	79%	periplasmic	
swp2088	Cytochrome c peroxidase	2	SO2178	33%	periplasmic	Cyt c5 peroxidase
swp2575	Cytochrome c oxidase cbb3-type, subunit III	2	S02361		Cyt Mem Pr	Donor to oxidase
swp2773	periplasmic nitrate reductase, cytochrome c-type protein	2	SO0845	43%	periplasmic	Donor to nitrate reductase
swp2866	Cytochrome-c peroxidase	2	SO2178	73%	periplasmic	Cyt c5 peroxidase
swp4011	cytochrome c, putative	2	SO0939	52%	periplasmic	
swp4047	thiol oxidoreductase	2	SO2178	25%	periplasmic	Cyt c5 peroxidase
swp4142	probable cytochrome-c peroxidase	2	SO2178	32%	periplasmic	Cyt c5 peroxidase
swp4458	Nitrate reductase cytochrome c-type subunit (NapB)	2	SO0845	59%	periplasmic	Donor to nitrate reductase
swp4554	cytochrome c family protein	2	SO4047	70%	periplasmic	
swp4555	Cytochrome c, class I	2	SO4048	59%	periplasmic	
Swp4608	Cytochrome c peroxidase	2	SO2178	42%	periplasmic	Cyt c5 peroxidase

swp4823	Cytochrome c oxidase, subunit II:Cytochrome c, class I	2	SO4606	64%	Cyt Mem Pr	Donor to oxidase
swp5075	soluble cytochrome cB	2	SO4666	74%	periplasmic	
swp0795	ubiquinol-cytochrome c reductase, cytochrome c1	1	SO0610	81%	Cyt Mem Pr	Quinol c5 reductase
swp2044	Cytochrome c, class I	1	SO0264	64%	periplasmic	Donor to oxidase and BCCP
swp2573	Cytochrome C oxidase, mono-heme subunit/FixO	1	SO2363	88%	Cyt Mem Pr	Donor to oxidase
swp3755	Cytochrome c, class II	1	SO3420	77%	periplasmic	
swp3853	Cytochrome c, class I	1	SO0714	58%	periplasmic	
swp3855	monoheme cytochrome c, putative	1	SO0716	59%	periplasmic	
swp3856	Cytochrome c, class I	1	SO0717	52%	periplasmic	
swp4146	putative cytochrome	1	SO4666	37%	periplasmic	
swp4577	cytochrome c family protein	1	SO4142	65%	periplasmic	

Table S5 Comparison of Cytochrome *c* categories in the WP3 and MR-1 genomes

gene categories	putative product	WP3	MR-1	differ
**1**	**decaheme cytochrome c**	**17**	**10**	**7**
2	Nitrate/TMAO reductases, membrane-bound tetraheme cytochrome c subunit	3	2	1
**3**	**fumarate reductase flavoprotein subunit precursor**	**3**	**7**	**-4**
4	Cytochrome c, class I	5	4	1
**5**	**nitrite reductase (cytochrome; ammonia-forming)**	**5**	**1**	**4**
6	cytochrome c oxidase, cbb3-type, subunit III	2	1	1
7	cytochrome c551 peroxidase	2	1	1
8	cytochrome c-type protein NapB	2	1	1
9	hypothetical protein	1	2	-1
10	cytochrome c'	1	1	0
11	cytochrome c family protein	1	1	0
12	cytochrome c oxidase, cbb3-type, subunit II	1	1	0
13	cytochrome c oxidase, subunit II	1	1	0
14	cytochrome c, putative	1	1	0
15	cytochrome c	1	1	0
16	monoheme cytochrome c, putative	1	1	0
other (no counterpart in the other genome)	cytochrome c, putative	8	6	2
	total	55	42	13

Table S6, WP3-specific genes (enzymes) in KEGG pathway

KEGG pathway map (total specific ORFs)	EC	specific genes
map00030:Pentose phosphate pathway, 5	1.1.99.10	swp3811
	1.1.99.3	swp0762, swp4566
	3.1.1.17	swp1560, swp2391
map00040:Pentose and glucuronate interconversions, 4	1.1.1.21	swp0549
	1.1.1.58	swp3368
	3.2.1.31	swp4524
	5.3.1.-	swp4289
map00051:Fructose and mannose metabolism, 4	1.1.1.21	swp0549
	1.1.99.21	swp4143, swp4566
	4.1.2.17	swp4629
map00052:Galactose metabolism, 4	1.1.1.21	swp0549
	2.7.7.10	swp4526
	2.7.7.12	swp4526
	3.2.1.108	swp1634
	3.2.1.23	swp1634, swp4524
	3.2.1.85	swp1634
map00053:Ascorbate and aldarate metabolism, 3	3.1.1.17	swp1560, swp2391
	4.1.2.20	swp0084
map00220:Urea cycle and metabolism of amino groups, 3	2.5.1.22	swp0820
	3.5.1.14	swp2054, swp3566
map00330:Arginine and proline metabolism, 9	1.2.1.19	swp2434, swp3810, swp4179
	1.4.3.3	swp1986
	2.5.1.22	swp0820

	3.5.3.11	swp3022
	3.5.3.7	swp3022
	4.3.1.12	swp3859
	5.1.1.4	swp1983, swp1985
map00361:gamma-Hexachlorocyclohexane degradation, 5	3.1.8.1	swp0131, swp1881, swp3662
	3.8.1.3	swp2700
	5.5.1.7	swp2107
map00410:beta-Alanine metabolism, 4	1.2.1.19	swp2434, swp3810, swp4179
	2.5.1.22	swp0820
map00500:Starch and sucrose metabolism, 10	2.4.1.12	swp2166
	2.4.1.14	swp1411
	2.4.1.20	swp4297
	2.7.7.33	swp1317, swp2168
	3.2.1.1	swp2674
	3.2.1.28	swp5044
	3.2.1.31	swp4524
	3.2.1.54	swp2674
	4.2.1.45	swp1598, swp3333
map00520:Nucleotide sugars metabolism, 3	2.7.7.10	swp4526
	2.7.7.12	swp4526
	2.7.7.33	swp1317, swp2168
map00530:Aminosugars metabolism, 3	4.1.3.3	swp1907, swp1984, swp2736
map00531:Glycosaminoglycan degradation, 5	3.1.6.12	swp1369, swp1721, swp2417
	3.1.6.13	swp1369, swp1721, swp2450
	3.1.6.14	swp2417, swp2450

	3.1.6.4	swp1369, swp1721, swp2417, swp2450
	3.2.1.23	swp1634, swp4524
	3.2.1.31	swp4524
map00561:Glycerolipid metabolism, 4	1.1.1.21	swp0549
	2.7.8.20	swp4846
	3.2.1.23	swp1634, swp4524
map00600:Glycosphingolipid metabolism, 6	3.1.6.1	swp1369, swp1721, swp2417, swp2450
	3.1.6.8	swp1369, swp1721, swp2417, swp2450
	3.2.1.23	swp1634, swp4524
map00930:Caprolactam degradation, 3	1.2.1.4	swp1982
	3.1.1.17	swp1560, swp2391
map00860:Porphyrin and chlorophyll metabolism, 3	1.3.1.33	swp1084
	3.2.1.31	swp4524
	5.4.1.2	swp0090

Table S7. WP3-specific genes involved in metabolism

**Gene**	**Length**	**Product**	COGid	COG
swp2594	371	Alanine dehydrogenase and pyridine nucleotide transhydrogenase	COG0686	E
swp0849	395	Alanine dehydrogenase/PNT, C-terminal:Alanine dehydrogenase/PNT, N-terminal	COG0686	E
swp1623	866	Glycoside hydrolase, family 3, N-terminal:Glycoside hydrolase, family 3, C-terminal	COG1472	G
swp1637	856	Glycoside hydrolase, family 3, N-terminal:Glycoside hydrolase, family 3, C-terminal	COG1472	G
swp1633	442	Sugar:cation symporter family protein	COG2211	G
swp4298	469	Sugar:cation symporter family protein	COG2211	G
swp1667	514	Phospholipase D/Transphosphatidylase	COG1502	I
swp2599	485	Phospholipase D/Transphosphatidylase	COG1502	I
swp3499	160	Ketosteroid isomerase homolog	COG4319	S
swp3501	126	Ketosteroid isomerase homolog	COG4319	S
swp2529	184	Acetyltransferase	-	-
swp1593	320	Acetyltransferase	-	-
swp2557	273	Acetyltransferase, GNAT family	-	-
swp1797	310	ATPase involved in DNA repair	-	-
swp2712	422	ATPase involved in DNA repair	-	-
swp1798	628	ATPase involved in DNA repair	-	-
swp1606	676	Bacterial sugar transferase/glycosyl transferase, WecB/TagA/CpsF family protein	-	-
swp0596	433	Conserved hypothetical exported protein	-	-

swp4872	194	Conserved hypothetical protein	-	-
swp0605	388	Conserved hypothetical protein	-	-
swp0332	395	Conserved hypothetical protein	-	-
swp0720	433	Conserved hypothetical protein	-	-
swp4034	444	Conserved hypothetical protein	-	-
swp1248	385	Glyceraldehyde 3-phosphate dehydrogenase	-	-
swp1245	464	Glyceraldehyde 3-phosphate dehydrogenase	-	-
swp3551	748	Omega-3 polyunsaturated fatty acid synthase PfaB	-	-
swp1612	443	Periplasmic protein involved in polysaccharide export	-	-
swp0036	248	Transcriptional regulator, AraC family	COG2207	K
swp0921	294	Transcriptional regulator, AraC family	COG2207	K
swp1415	332	Transcriptional regulator, AraC family	COG2207	K
swp2096	303	Transcriptional regulator, AraC family	COG2207	K
swp2451	302	Transcriptional regulator, AraC family	COG2207	K
swp2963	134	Transcriptional regulator, AraC family	COG2207	K
swp3419	311	Transcriptional regulator, AraC family	COG2207	K
swp4166	343	Transcriptional regulator, AraC family	COG2207	K
swp4561	247	Transcriptional regulator, AraC family	COG2207	K
swp4627	274	Transcriptional regulator, AraC family	COG2207	K
swp4817	353	Transcriptional regulator, AraC family	COG2207	K
swp4383	300	Response regulator receiver	-	-
swp4661	487	TPR repeat:Transcriptional regulatory protein, C-terminal	COG0457	R
swp2993	512	Transcriptional activator CadC	-	-
swp4848	662	Transcriptional regulatory protein, C-terminal	-	-
swp2124	699	Transcriptional regulatory protein, C-terminal	-	-

swp3288	740	Transcriptional regulatory protein, C-terminal	-	-
swp3840	711	Transcriptional regulatory protein, C-terminal	COG1506	E
swp4661	487	TPR repeat:Transcriptional regulatory protein, C-terminal	COG0457	R
swp4504	510	C-5 cytosine-specific DNA methylase	COG0270	L
swp0839	475	Site-specific DNA-methyltransferase	COG0270	L
swp0008	412	Site-specific DNA-methyltransferase (cytosine-specific)	COG0270	L
swp0042	353	Potassium transport system, Trk family	COG0492	O
swp0991	350	Potassium transporter (Trk family)	COG2072	P
swp3342	662	BCCT transporter	COG1292	M
swp3813	405	Conserved hypothetical transmembrane protein	COG1292	M
swp3320	525	Glycine betaine transporter	COG1292	M
swp4938	518	Probable carnitine transporter	COG1292	M
swp1561	312	Glycosyl transferase, family 2	COG0463	M
swp1569	289	Glycosyl transferase, family 2	COG0463	M
swp1607	359	Glycosyl transferase, family 2	COG0463	M
swp2920	393	Glycosyl transferase, family 3	COG0547	E
swp4044	316	Glycosyl transferase, family 4	COG0438	M
swp1411	386	Glycosyl transferase, family 4	COG0438	M
swp0660	348	Glycosyl transferase, putative	COG0463	M
swp4772	228	Glycosyltransferase, putative	-	-
swp2166	619	Glycosyltransferases, probably involved in cell wall biogenesis	COG1215	M

Table S8. WP3-specific genes involved in sulfur metabolism.

Gene	Product	COG	son	ppr	cps	vpa	ilo	pha
swp0240	Sodium/sulphate symporter	COG0471	-	+	+	+	+	+
swp1591	Putative transporter	COG0471	-	+	+	+	+	+
swp3241	Sodium/sulphate symporter	COG0471	-	+	+	+	+	+
swp4056	TrkA-C	COG0471	-	+	+	+	+	+
swp1034	Arylsulfatase regulator (Fe-S oxidoreductase)	COG0641	-	+	+	+	-	-
swp1718	Arylsulfatase regulator (Fe-S oxidoreductase)	COG0641	-	+	-	+	-	-
swp2412	Arylsulfatase regulator (Fe-S oxidoreductase)	COG0641	-	+	-	+	-	-
swp5020	Arylsulfatase regulator (Fe-S oxidoreductase)	COG0641	-	+	+	+	-	-
swp1369	Sulfatase family protein	COG3119	-	+	+	+	-	-
swp1721	Arylsulfatase A	COG3119	-	+	+	+	-	-
swp2417	Sulfatase family protein	COG3119	-	+	+	+	-	-
swp2450	Probable sulfatase	COG3119	-	+	+	+	-	-
swp1883	Glutathione S-transferase, N-terminal	COG0625	-	+	+	-	-	-
swp3308	Glutathione S-transferase, C-terminal	COG0625	-	+	+	-	-	-
swp4375	Glutathione S-transferase	COG0625	-	+	-	-	-	-
swp5099	Glutathione S-transferase	COG0625	-	+	-	-	-	-

Abbreviations: son, *Shewanella oneidensis*; ppr, *Photobacterium profundum*; cps, *Colwellia psychrerythraea*; vpa, *Vibrio parahaemolyticus*; ilo, *Idiomarina loihiensis*; pha, *Pseudoalteromonas haloplanktis*.

+: present; -: absent

Table S9. WP3 genes involved in tRNA, rRNA modifications

Gene	Length	Product	COGid	COG
swp3116	261	tRNA pseudouridine synthase	COG0101	J
swp1220	316	tRNA pseudouridine synthase B	COG0130	J
swp3267	259	tRNA--hydroxylase	COG4445	FJ
swp3299	281	Pseudouridine synthase	COG0564	J
swp4598	319	Pseudouridine synthase	COG0564	J
swp4996	237	Pseudouridine synthase RluA-like	COG0564	J
swp3055	308	Pseudouridine synthase, RluD	COG0564	J
swp3940	328	Pseudouridine synthase, RluD	COG0564	J
swp1672	217	Pseudouridylate synthase	COG0564	J
swp3530	278	Pseudouridylate synthase	COG0564	J
swp4339	225	Pseudouridylate synthase	COG0564	J
swp2518	256	Pseudouridylate synthases, 23S RNA-specific	COG0564	J
swp0893	233	Pseudouridine synthase, Rsu	COG1187	J
swp1866	226	Pseudouridine synthase, Rsu	COG1187	J
swp1929	290	Pseudouridine synthase, Rsu	COG1187	J
swp3865	230	Pseudouridine synthase, Rsu	COG1187	J
swp4417	240	Pseudouridine synthase, Rsu	COG1187	J
swp4126	228	Ribosomal small subunit pseudouridine synthase A, putative	COG1187	J
swp4290	210	RNA pseudouridine synthase family	COG1187	J

protein
